# Salivary Gland Cancers in the Era of Molecular Analysis: The Role of Tissue and Liquid Biomarkers

**DOI:** 10.3390/cancers17040660

**Published:** 2025-02-15

**Authors:** Elisabetta Broseghini, Francesca Carosi, Mirea Berti, Samuele Compagno, Anna Ghelardini, Matteo Fermi, Giulia Querzoli, Daria Maria Filippini

**Affiliations:** 1 IRCCS Azienda Ospedaliero—Universitaria di Bologna, 40138 Bologna, Italy; elisabett.broseghin2@unibo.it; 2 Division of Medical Oncology, IRCCS Azienda Ospedaliero—Universitaria Sant’Orsola Malpighi, 40138 Bologna, Italy; francesca.carosi3@studio.unibo.it (F.C.); mirea.berti@studio.unibo.it (M.B.); samuele.compagno@studio.unibo.it (S.C.); anna.ghelardini@studio.unibo.it (A.G.); 3 Department of Otorhinolaryngology—Head and Neck Surgery, IRCCS Azienda Ospedaliero—Universitaria di Bologna, 40138 Bologna, Italy; matteo.fermi3@unibo.it; 4 Pathology Unit, IRCCS Azienda Ospedaliero Universitaria di Bologna, 40138 Bologna, Italy; 5 Department of Medical and Surgical Sciences (DIMEC), Alma Mater Studiorum, Università di Bologna, 40138 Bologna, Italy

**Keywords:** salivary gland cancers, head and neck cancer, molecular analysis, liquid biopsy, biomarkers, targeted therapy

## Abstract

**Background**: Salivary gland cancers (SGCs) are a rare and heterogeneous group of malignancies, accounting for approximately 5% of head and neck cancers. Despite their rarity, advances in molecular profiling have revealed a variety of genetic and molecular pathways, many of which are potentially actionable with targeted therapies. **Methods**: We reviewed the current literature involving the molecular landscape of SGCs, encompassing the diagnostic and prognostic value of tissue and liquid biomarkers and the potential therapeutic targets across various histological subtypes. **Results**: Our review highlights key molecular diagnostic findings such as the *CRTC1-MAML2* fusion in mucoepidermoid carcinoma and *MYB-NFIB* rearrangements in adenoid cystic carcinoma, but also targetable alterations such as HER2 and AR positivity in salivary duct carcinoma and *ETV6-NTRK3* fusion in secretory carcinoma. Liquid biopsy (both blood- or salivary-based), including circulating tumor DNA, circulating tumor cells, and miRNAs, offers novel, noninvasive approaches for disease monitoring and personalized treatment. Emerging therapies such as HER2 inhibitors, androgen deprivation therapy, and TRK inhibitors underscore the shift towards precision oncology in managing these malignancies. **Conclusions**: Despite promising advances, challenges remain due to the rarity and phenotypic heterogeneity of SGCs, emphasizing the need for molecularly stratified clinical trials. This review presents an overview of tissue and liquid biomarkers, focusing on molecular targets and therapeutic innovations that lay the foundation for improved diagnostic and treatment strategies for SGCs.

## Simple Summary

Salivary gland cancers are rare but can be aggressive, making early detection and effective treatment crucial. Understanding the genetic and molecular alterations opens the door for targeted treatments that can be more effective than traditional therapies. The review discusses the molecular landscape of different salivary gland cancer subtypes, including their diagnostic biomarkers and potential therapeutic targets. For example, HER2 and androgen receptor positivity in salivary duct carcinoma and the ETV6-NTRK3 fusion in secretory carcinoma are important for treatment planning. New methods, like liquid biopsies, offer a minimally invasive approach to monitor tumor dynamics and assess treatment response. These biomarkers can aid in early diagnosis, prognosis, and differentiation between malignant and benign tumors. However, the rarity and heterogeneity of these cancers pose challenges in treatment and require specific molecularly stratified clinical trials to improve patient outcomes.

## 1. Introduction

Salivary gland cancers (SGCs) are a rare and complex group of neoplasms, representing approximately 5% of head and neck cancers (HNCs) in Europe. Globally, their annual incidence ranges from 0.57 to 0.69 cases per 100,000 individuals, with a slight male prevalence and a higher rate of diagnosis in the sixth–seventh decades of life [1,2]. The broad histological diversity of these tumors, including over 20 recognized malignant subtypes classified by the World Health Organization (WHO), leads to heterogeneous biological behaviors, prognoses, and therapeutic implications, underscoring the importance of a detailed histopathological and molecular characterization [3].

The integration of molecular diagnostics, including fluorescence in situ hybridization (FISH), reverse transcription–polymerase chain reaction (RT-PCR), and next-generation sequencing (NGS), has significantly implemented an accurate classification of SGCs. These tools are not only helpful for making accurate histological diagnoses but also for identifying actionable mutations, which allow for the use of targeted therapies [4].

The histologic grade is a strong predictor of outcome: low- and intermediate-grade SGCs have a 5-year survival of 85–90%, while high-grade SGCs report less than 40% [5,6].

Tumor size, nodal status, the presence of extracapsular spread, and margin status are additional predictors of poor outcomes [6].

The localized/locoregional disease is managed with surgery with or without post-operative radiation therapy (RT) in case of advanced T stage (T3-T4 disease), high/intermediate grade, incomplete resection margins, perineural invasion, and nodal involvement [7]. Retrospective series tried to establish a role for adjuvant chemotherapy in addition to RT, but with no apparent clinical benefit, even if high-risk features were present [7].

More than 70% of patients develop a recurrent/metastatic (R/M) disease, varying in incidence among different histological subtypes [8]. Conventional chemotherapy is mostly indicated, although the rarity of these conditions and their heterogeneity hinder large randomized clinical trials. Moreover, the majority of studies included all SGC subtypes, and we do not have information about the efficacy of each treatment in the specific histopathological subtype [9,10].

Our review highlights the molecular landscape of SGCs, encompassing the diagnostic and prognostic value of tissue and liquid biomarkers and the potential therapeutic targets across various histological entities.

## 2. Tissue Biomarkers Across Various Salivary Gland Subtypes: Diagnostic and Prognostic Roles

In the following sections, we provide an overview of the specific tissue markers and molecular alterations associated with selected SGC subtypes, focusing on their diagnostic utility, prognostic value, and potential as therapeutic targets. Not every histological subtype has been included despite exhibiting distinctive molecular alterations.

Table 1 provides a summary of recurrent immunohistochemical tumor markers, detectable mutations and molecular alterations observed in SGC subtypes.

### 2.1. Mucoepidermoid Carcinoma (MEC)

Mucoepidermoid carcinoma (MEC) is the most common tumor type, accounting for 10–15% of SGCs and commonly occurring in the parotid gland, especially the high-grade type [11]. MEC is traditionally characterized as a “triphasic” tumor consisting of varying proportions of mucus-producing cells (which contribute the “muco” in “mucoepidermoid”), epidermoid (squamous-like) cells (responsible for the “epidermoid” component), and intermediate cells. Additionally, columnar cells are often present, and all these cell types may undergo clear cell or oncocytic changes. In most cases, MEC can be easily diagnosed based on routine histological examination alone, without the need for additional testing [12]. The presence of immunohistochemical p63 or p40 expression, combined with the absence of S100/SOX10 staining, can assist in distinguishing MEC from other SGCs [13].

Some studies have suggested that elevated MUC-1 expression may serve as a potential indicator of poor prognosis in high-grade MEC and could be explored as a molecular target to enhance patients’ outcomes in the future [14,15]. Moreover, high expression of HER2 and EGFR is typical of high-grade tumors, reported in 45% and 14% of cases, respectively, and is associated with a poor prognosis [16]. Amphiregulin (AREG), an EGFR ligand, has been shown to be a downstream target of *CRTC1-MAML2* fusion and, when overexpressed in immunohistochemistry (IHC), showed a correlation with longer disease-free survival [17].

High-grade MECs often harbor additional molecular alterations, including *TP53* mutations, which are linked to worse outcomes [18].

Most MEC are characterized by a translocation t(11;19)(q14-21;p12-13) resulting in *CRTC1-MAML2* oncogene fusion [19]. The other translocation, t(11;19)(q21;q26), leads to a *CRTC3-MAML2* fusion product that is detected in 6% of cases [20].

An additional uncommon alteration is the t(6;22)(p21;q12) translocation, which drives the formation of the *ESWR1–POU5F1* fusion [21] and is associated with a favorable prognosis in low-grade MECs. The detection of these rearrangements may provide information to distinguish the oncocytic variant of MEC from oncocytoma and oncocytic carcinoma [22] or the “Warthin-like” MEC from the Warthin tumor [23].

*CRTC1-MAML2* and *CRTC3-MAML2* fusions appear to be associated with low-grade tumors, favorable clinical behavior, younger age (<60 years), and better prognosis [24,25]; on the other hand, fusion-negative tumors present a more aggressive biological behavior, characterized by coexisting *TP53* mutations [25,26]. *CDKN2A* deletions in *CRTC1-MAML2* fusion-positive MECs have been associated with an unfavorable prognosis [27].

Recently, microRNA have been described in MEC: the miR-17-92 cluster is upregulated in MEC with a poor prognosis [28], and miR-205-5p and miR22 upregulation, increasing cellular invasion potential, are linked to worse OS rates [29]. Conversely, the upregulation of miR-34a, involved in the suppression of c-Kit and β-catenin, is associated with improved OS [30]. Furthermore, specific miRNAs were tied to MEC grading, such as miR-4324, which is significantly lower in high-grade MEC and correlates with poorer OS [29].

### 2.2. Adenoid Cystic Carcinoma

Adenoid cystic carcinoma (ACC) accounts for approximately 10% of SGCs and ranks as the second most prevalent malignancy in these glands, following MEC [31]. It is the most common tumor of minor salivary glands. While ACC is recognized as a histopathological subtype with slow growth, it has a propensity for recurrence, often exhibiting perineural invasion and distant metastases, particularly to the lungs [32]. ACC is a biphasic epithelial tumor comprising myoepithelial and ductal cells, and due to its biological variability, it remains a challenging diagnosis [33]. Ductal cells are characterized by eosinophilic cytoplasm and uniformly round nuclei (positive for CK7 and CAM 5.2), while myoepithelial cells exhibit clear cytoplasm and hyperchromatic, angular nuclei (positive for calponin, p63, SOX10, S100, and SMA). The overexpression of c-Kit (CD117) is restricted to inner epithelial cells, and it is a prognostic factor of aggressive behavior, development of distant metastasis, and worse survival outcome [34,35]. MYB protein through IHC is widely accessible but does not offer optimal specificity [36].

Alterations in PI3K/AKT/mTOR, *TP53* and *TERT* promoter mutations have been reported [37].

Furthermore, *NOTCH* mutations are associated with an aggressive subgroup of ACC, characterized by a higher rate of liver and bone metastasis, shorter relapse-free survival, and OS compared to *NOTCH* wild-type ACC [38].

Ho et al. also observed common alterations in genes involved in chromatin remodeling in R/M ACCs, including *KDM6A*, *KMT2C/MLL3*, *ARID1A*, *ARID1B*, *BCOR*, *MLL2/KMT2D*, and *CREBBP*, with a higher frequency compared to primary tumors [39].

Rearrangements involving MYB or MYBL1 are highly specific for ACC and constitute valuable diagnostic hallmarks, usually identified with FISH or NGS, not only for salivary gland ACC but also for ACC arising in other anatomic sites (lung, breast, trachea, sinonasal cavity, lacrimal glands, and skin) [40,41,42]. *MYB* is an oncogene controlling proliferation and differentiation acting as a DNA-binding transcription regulator and is not observed in normal salivary gland parenchyma [43]. Most ACC present a *MYB-NFIB* fusion, but in a not negligible percentage of cases, other chromosomal translocations have been observed [38].

Regarding miRNA, recent evidence showed that miR-6835-3p, miR-4676, and miR-1180 are predictive factors of reduced survival rates [44], and another study associated miR-20a and miR-17 with poor outcomes [38]. Additionally, miRNA profiles in ACC revealed altered expressions of miR-4487, miR-4430, miR-486-3p, miR-5191, miR-3131, and miR-211-3p during metastatic progression [45]. MiR-582-5p downregulation has been linked to reduced invasion and migration in ACC [46].

### 2.3. Acinic Cell Carcinoma

Acinic cell carcinoma (AciCC) is generally considered a low-grade tumor with an overall favorable prognosis, but a high recurrence rate has been reported based on the presence of poor prognostic factors. AciCC is usually positive for CK7 and CAM 5.2 and negative for S100. DOG1, well known for its expression in gastrointestinal stromal tumors (GIST), can be detected by IHC to identify well-differentiated AciCC; in fact, poorly differentiated tumors show a moderate to poor expression of DOG1 [47,48]. Moreover, AciCC is usually immunonegative for mammaglobin, which is useful in its distinction from secretory carcinoma.

Genetic profiling in AciCC assessed that *CDKN2A* and *CDKN2B* mutations are strongly associated with the presence of metastatic or relapsed disease (up to 90% of cases) and also with high-grade tumors (nearly 60%), highlighting them as a negative prognostic indicator [49,50]. Additionally, other genetic alterations have been observed in advanced AciCC: the most common rearrangements involved *ATM*, *PTEN*, *FBXW7*, and *TP53*, whereas *BRAF*, *NF1*, *HRAS*, *NOTCH1*, *TERT*, *ARID2*, *BIRC3*, *MTAP*, and *FAT1* mutations were less frequent [49,50].

Haller et al. identified the t(4;9)(q13;q31) rearrangement, which leads to the fusion of the secretory Ca-binding phosphoprotein (SCPP) gene cluster (including *STATH*, *HTN1*, *HTN3*, *ODAM*, *FDCSP*, and *MUC7*) with the *NR4A3* gene (in 80% of cases), which acts as an oncogenic driver. This particular translocation is exclusive to AciCC, helping differentiate it from secretory carcinoma, especially in high-grade transformation cases [51].

The second most frequent fusion involves the *HTN3* and *MSANTD3* genes (t(4;9)(q13.3;q31.1)), which has been observed in a small number of cases (4–8%) [52,53,54]. Recent molecular studies have identified *PON3-LCN1* fusion in a subset of AciCC cases. While this fusion is not yet targetable, its presence serves to differentiate AciCC from other SGCs [55].

### 2.4. Salivary Duct Carcinoma

Salivary duct carcinoma (SDC) is an aggressive epithelial tumor originating from intralobular and interlobular excretory ducts. Histologically, it mostly resembles invasive ductal breast carcinoma (DBC) but with marked cell atypia and high mitotic count and is frequently associated with distant metastasis [54]. CK7 shows consistent positivity, while S100 and SOX10 are negative. Staining for p63 can assist in identifying the intraductal component by highlighting the basal/myoepithelial cells around the neoplastic cells [56,57,58].

**Figure 1:**
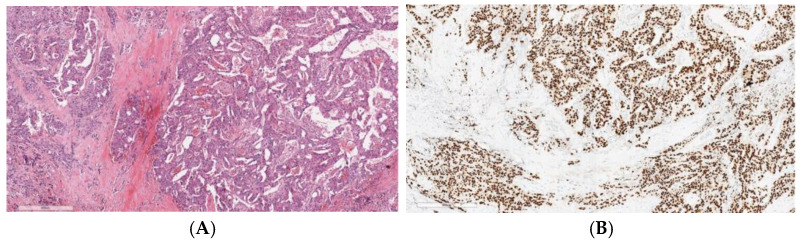
Invasive salivary duct carcinoma: (**A**) Histological image stained with hematoxylin and eosin of an invasive salivary duct carcinoma, showing a complex architecture with cribriform gland formation, a Roman-bridge pattern, and solid nests. The tumor cells display large, pleomorphic nuclei with coarse chromatin, prominent nucleoli, and abundant eosinophilic cytoplasm, which is typically apocrine in nature. (**B**) Strong immunoreactivity of the neoplastic cells for androgen receptors.

Approximately 70% of these tumors are androgen receptor (AR)-positive, revealed by IHC staining [59] (Figure 1). About 30% of SDC present HER2 overexpression by IHC or HER2 amplification by FISH or next-generation sequencing (NGS) [59], both associated with poor prognosis. These alterations are more frequently observed in SDCs ex-pleomorphic adenoma (SDC ex-PA) [60].

Notably, while *HRAS* mutations are prevalent in de novo lesions, they are rare in SDC ex-PAs.

In recent years, there has been significant progress in understanding the genetics of SDC, although it has yet to be thoroughly explored. The tumor mutation burden is notably high in most SDC cases compared to other SGCs. Genetic fusions are not commonly observed in this subtype, but somatic mutations are significantly more frequent [61,62]. The most common alterations include *PIK3CA*, *HRAS*, *NRAS*, *BRAF*, *EGFR*, *AKT1,* and *ERBB2* (HER2) rearrangements, some of which are associated with poor prognosis [56,61,62,63].

### 2.5. Other Subtypes

**Figure 2:**
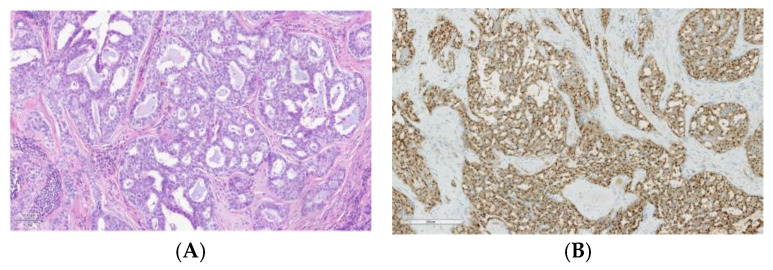
Secretory carcinoma: (**A**) Histological image stained with hematoxylin and eosin of a secretory carcinoma showing a lobulated growth pattern, divided by fibrous septa, and consists of a combination of microcystic, tubular, follicular, and papillary-cystic structures containing characteristic luminal secretions. The tumor cells feature low-grade, round to oval nuclei with finely granular chromatin and prominent central nucleoli. The cytoplasm is pale pink with a granular or vacuolated appearance. Cellular atypia is generally mild, and mitotic activity is absent. (**B**) Strong nuclear staining for pan-TRK.

Secretory carcinoma (Figure 2A), previously known as mammary analog secretory carcinoma (MASC), predominantly arises from the parotid gland. ETV6-NTRK3 gene translocation t(12:15)(p13;q25) has been identified in the majority of secretory carcinoma and comes to be considered as a distinctive molecular feature, helpful as an additional diagnostic marker and providing a therapeutic target [64]. Consequently, IHC is frequently positive for pan-TRK (Figure 2B), which makes it a great biomarker to differentiate secretory carcinoma from other low-grade SGCs [65].

Even if *ETV6-NTRK3* represents the most common rearrangement, additional *ETV6* fusion partners have been identified, such as *ETV6-MAML3* [66], *ETV6-MET* [67], and *ETV6-RET* [68].

*NTRK* gene fusions are important driver mutations that have led to the development of therapeutic agents [69].

Polymorphous adenocarcinoma (PAC) is a low-grade tumor primarily affecting minor salivary glands, and it can be accurately diagnosed using morphology and immunophenotype. Typical markers include diffuse positivity for CK-7, S100, CEA, GFAP, and SOX10, patchy expression of p63, and typical negativity for p40 [70]. PAC consists of a single cell type, likely representing a progenitor cell that precedes both luminal and myoepithelial differentiation. These cells originate from the intercalated duct and exclusively express DOG1, particularly in areas where lumen formation occurs and in the apical region of the cell [71].

Most PACs are associated with *PRKD1* mutations. These mutations involve hotspot alterations leading to constitutive activation of protein kinase D1, which promotes tumor cell proliferation. PRKD1 mutations are highly specific to PAC and can aid in differentiating it from other low-grade subtypes [72]. Interestingly, in cribriform adenocarcinoma (CA), *PRKD1-3* fusions are the most frequent [73]. CA is considered an aggressive variant of PAC, with a high likelihood of nodal metastasis at presentation. Among the fusion partners, *ARID1A*, *ATL2*, *DDX3X*, *PPP2R2A*, *PRKAR2A*, *SNX9*, and *STRN3* (in cases with high-grade transformation) are particularly noteworthy [74,75,76]. However, the type of genomic alteration is not exclusive to any specific adenocarcinoma subtype [77].

Hyalinizing clear cell carcinoma (HCCC) is a malignant epithelial tumor of salivary gland origin with squamous differentiation, most commonly arising in the minor salivary glands of the oral cavity and the base of the tongue. HCCC typically presents as infiltrative cords of cells with eosinophilic to clear cytoplasm and round to raisinoid nuclei set within a prominent fibroblastic or hyalinized stroma [78]. The tumor exhibits a squamoid immunophenotype, usually showing diffuse positivity for p40 and CK5/6, though mucin production may also be observed. Some tumors express p16, which can create a diagnostic challenge when the tumor originates in the oropharynx; however, they do not contain transcriptionally active HPV. Myoepithelial markers such as S100, SMA, and calponin are negative. Most HCCC cases are characterized by the EWSR1::ATF1 fusion, which is also found in clear cell sarcoma [79]. The identification of this fusion has established HCCC as a distinct entity rather than a catch-all category for carcinomas with clear cells. Due to consistent breakpoints, EWSR1 FISH is a reliable and widely available method for confirming HCCC. A subset of cases is associated with the *EWSR1::CREM* fusion [80]. Identifying an *EWSR1* fusion can definitively confirm the diagnosis when squamous cell carcinoma is part of the differential diagnosis.

Intraductal carcinoma (IDC). IDC most commonly occurs in the parotid gland and has been referred to by various names, such as low-grade salivary duct carcinoma and low-grade cribriform cystadenocarcinoma [81]. Initially, IDC was thought to represent a noninvasive neoplasm, similar to breast ductal carcinoma in situ, based on its benign behavior and histopathological features, which mirrored those of breast ductal carcinoma in situ—proliferating ducts filling rounded spaces and encased by a continuous layer of flattened myoepithelial cells. However, the current perspective is more nuanced [82]. The IDC category encompasses four distinct, though sometimes overlapping, subtypes. Intercalated duct IDC is characterized by small amphophilic to eosinophilic cells with oval nuclei. Apocrine IDC consists of large cells with bubbly eosinophilic cytoplasm, often displaying snouting and decapitation secretions. Oncocytic IDC features cells with abundant granular eosinophilic cytoplasm and round nuclei with prominent nucleoli, while mixed IDC presents a combination of these characteristics. Intercalated duct and oncocytic IDCs typically exhibit low-grade morphology with minimal mitotic activity, whereas apocrine IDC can range from low to high grade. The surrounding tissue frequently shows degen-erative alterations, including fibrosis, inflammation, cholesterol clefts, and hemorrhage. Invasive growth with the loss of myoepithelial cells may occasionally be observed in all IDC subtypes.

It is now understood that a significant portion of IDC cases harbor gene fusions. The most common fusion is *NCOA4::RET*, which can occur in any IDC subtype [83]. *TRIM33::RET* is primarily found in oncocytic IDC, while TRIM27::RET is associated with mixed types [84]. Although these are the most frequently identified fusions, the list of known fusions is expanding (Table 1). It is increasingly evident that fusion-positive IDCs are not carcinoma in situ but rather biphasic tumors [12].

Epithelial–myoepithelial carcinoma (EMC) is composed of two distinct cell populations that form a double-layer structure: inner ductal cells and outer myoepithelial cells. EMC can present in various histological subtypes, including sebaceous, oncocytic, and double-clear, which can complicate the differential diagnosis [85]. EMC is more common in females than in males, with the parotid gland and submandibular gland being the most frequently affected site. Typically, it presents as a slow-growing, painless mass. Histologically, low-molecular-weight cytokeratins are strongly positive in the ductal component and are less intense in the myoepithelial component. Myoepithelial markers include SMA, HHF35, p63, and calponin. S100 stains both the myoepithelial and the ductal components.

*HRAS* mutations (27–87%) are the most frequently observed genetic alterations in EMC [86], although they were not found in EMCs arising from pleomorphic adenomas (PAs) [87]. Molecular alterations in *PIK3CA* and *AKT1* are relatively common in EMC [88].

Carcinoma ex-pleomorphic adenoma (CA ex PA) is a rare form of primary SGC that develops from a pre-existing PA. It is estimated that 5–15% of benign PAs undergo malignant transformation into carcinoma [89]. The presence of benign tumor components can sometimes lead to misdiagnosis, though rapid tumor growth and other associated symptoms should raise suspicion of malignancy. While the most common malignant components of Ca ex PA are SDC, myoepithelial carcinoma (MECA), and adenocarcinoma not otherwise specified (NOS), other types of SGCs have also been identified. Genetic alterations in the pleomorphic adenoma gene 1 (*PLAG1*) and the high-mobility group AT-hook 2 (*HMGA2*) genes are frequently seen in both PAs and Ca ex PAs, although these alterations are not typical of primary SDC, MECA, or adenocarcinoma NOS [90,91]. The alterations reported in the SDC subtype are amplification in *HMGA2*, *MDM2*, and *ERBB2* (HER2) [92].

Myoepithelial carcinoma (MECA) accounts for only 2% of all SGCs, and most cases occur in the parotid gland. MECA has a propensity for distant metastases. It can either occur as a de novo tumor or result from the malignant transformation of a pre-existing PA or myoepithelioma [93]. Studies suggest that MECA arising from PAs is more commonly detected than de novo cases [94]. However, it remains unclear which form exhibits more aggressive behavior or poorer outcomes. Due to the rarity of salivary gland MECA, limited genetic studies have been conducted. Dalin et al. analyzed 40 tumors, dividing them into MECA de novo and MECA ex PA, as well as cases with and without recurrence. They found that MECA ex PA tumors exhibited more genetic alterations, including fusions, somatic mutations, and copy number variations (CNVs). The authors suggested that CNVs are involved in the malignant transformation of PA into MECA ex PA and are associated with worse prognosis [95]. The most common fusion identified in MECA ex PA was *PLAG1-FGFR1* (18%), followed by *PLAG1- TGFBR3*, though these fusions did not show prognostic significance. *EWSR1-ATF1* was found only in de novo MECA cases, with or without recurrence. *EWSR1* rearrangements were frequently seen in the clear cell component of MECA [94,95].

## 3. Liquid Biopsy for SGCs: Potential Clinical Applications and Future Perspectives

**Figure 3:**
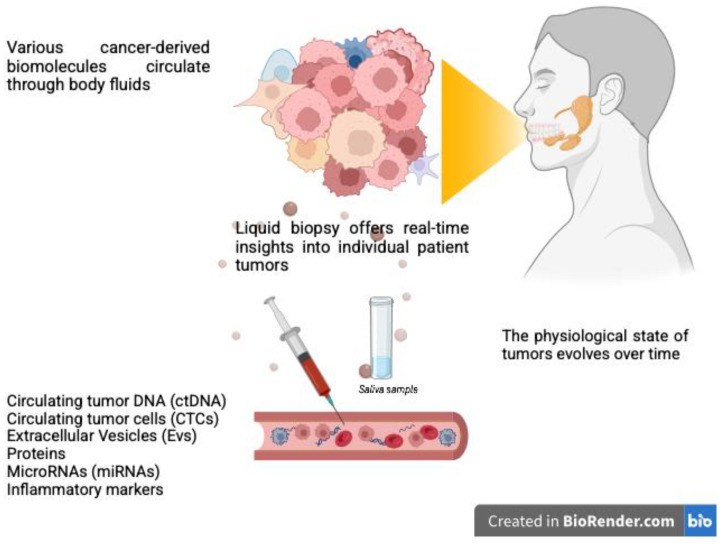
Liquid biopsy for salivary gland cancer diagnosis and monitoring involves detecting cancer-derived biomaterials released into body fluids, such as blood and saliva. These biomolecules act as critical indicators, potentially providing real-time insights into the disease status. This innovative approach could support personalized treatment strategies for patients with salivary gland cancers. Created with BioRender.com.

Liquid biopsy is the term used to refer to several technologies which enable the molecular analysis of bodily fluids (most commonly peripheral blood, but also pleural and peritoneal fluids, urine, saliva, and cerebrospinal fluid) and permit early diagnosis, prognosis assessment, and acquisition of information about tumor behavior and response to therapy [96]. In recent years, it has been shown to offer several advantages in medical oncology, including the capacity to overcome the limitations of traditional tissue biopsy through a minimally invasive and quick approach. Among the various biomarkers, the most studied are represented by circulating tumor DNA (ctDNA), circulating tumor cells (CTCs), extracellular vesicles (EVs), and circulating microRNAs (miRNAs) (Figure 3).

Here, we provide an overview of liquid biopsy biomarkers that have been studied for SGCs. In the mentioned studies, SGCs were often examined as a whole, without specification according to histotype and/or in association with other HNCs.

ctDNA consists of a small fraction of cell-free DNA fragments ranging from 160 to 2000 base pairs (bp), which is released into bodily fluids from the primary tumor following tumor cell death or via active secretion and is characterized by tumor-specific gene rearrangements, as the one expressed in the originating tumor, potentially leading to the identification of targetable mutations. It is relatively easy to extract and can be quantified accurately using the appropriate techniques, even during the early stages of cancer [97,98]. Typically, ctDNA is more fragmented than extracellular DNA (with fragments around 90–150 bp in length) [99], and its half-life in peripheral blood ranges from 15 min to several hours, making it an ideal biomarker for real-time monitoring of tumor dynamics [97]. ctDNA levels had been investigated as a biomarker to predict radiological response in patients with SDCs from the CABO-ASAP trial. The results suggested a potential correlation between changes in ctDNA levels and radiological response in these patients. At baseline, ctDNA was detectable in four out of five patients, with levels varying significantly between individuals, highlighting the dynamic nature of ctDNA shedding by tumors. Notably, in two patients, an increase in ctDNA was detected before any radiological or clinical signs of disease progression, demonstrating the potential of ctDNA for early monitoring of disease progression [100].

CTCs are malignant cells that detach from primary tumors and circulate as individual cells or in clusters; in the bloodstream, they can travel to distant tissues and establish metastasis [101]. The presence and number of CTCs are emerging as potential predictors of recurrence [102]. CTCs also have utility for in vitro studies to conduct functional tests, including drug testing or creating experimental models like xenografts [103,104]. However, one major challenge of using CTCs in liquid biopsy is their low abundance in the bloodstream—usually only 1–10 cells per 10 mL of blood—which makes their detection and isolation technically difficult [97].

CTCs have been recognized as a potential marker for early metastasis, treatment response, and surveillance in HNCs [105,106], including SGCs. In a pilot study involving patients with ACC, CTCs were detected in three patients (3/8, 37.5%), all of whom had either recurrent local disease or known distant metastatic disease [107]. Additionally, cultures derived from CTCs closely mirror the histological and molecular characteristics of uncultured ex vivo samples. Notably, the generation of models suitable for small-scale drug screening took less than three months, a timeframe that could provide valuable treatment-relevant information for patients, although larger studies are required to systematically assess the effectiveness of CTC expansion in SGCs and the correlation between in vitro drug responses and patients’ outcomes [108].

EVs are membrane-bound vesicles secreted in various bodily fluids, also by cancer cells, and are crucial for intercellular communication [109,110]. They are classified into three types based on their size and origin: exosomes (40–150 nm), microvesicles (40–1000 nm), and apoptotic bodies (800–5000 nm). Among these, exosomes are the most studied due to their stability in circulation, ability to penetrate tissues, and role in promoting tumor progression [111,112,113,114]. Exosomes contain a variety of biomolecules, including RNAs and proteins, which can mediate oncological processes and support tumor growth, particularly in pre-metastatic niches. These exosomal RNAs include not only protein-coding RNAs but also regulatory miRNAs, long non-coding RNAs (lncRNAs), and small nucleolar RNAs (snoRNAs), which contribute to cancer progression [115,116,117]. Moreover, exosomes in SGCs are involved in tumor progression by tumor microenvironment modulation, immune response regulation, and promotion of metastasis [118].

Inflammatory markers have also been examined to distinguish parotid gland tumors from healthy controls [119]. IL-33 is a cytokine with a dual function, currently investigated along with its receptor, sST2, because of its association with negative prognosis in various cancer types, and it has also been identified in SGCs tissue; it has been observed that the serum IL-33 level was significantly elevated in patients with parotid gland tumors, and sST2 receptor levels were significantly higher in benign neoplasms such as pleomorphic adenoma but also in AciCC patients compared to the controls [119]. Another study identified significantly elevated carbohydrate antigen 19-9 (CA 19-9) in saliva from malignant parotid gland tumor cases, showing CA 19-9 as a new diagnostic tool in the preoperative differentiation between malignant and benign parotid tumors [120].

A study was conducted to assess the levels of carcinoembryonic antigen (CEA) and carcinoma-associated antigen-50 (CA-50) in patients with oral cancers and SGCs. Concentrations of salivary CEA and CA-50 were measured in 80 patients with these types of tumors, 40 patients with benign tumors, and 80 healthy controls. The results showed that measurements of salivary CEA and CA-50 were more sensitive than serum measurements. Salivary levels of both CEA and CA-50 were significantly higher in patients with malignant tumors compared to those with benign tumors and healthy controls (*p*-value < 0.001). In contrast, only 7 and 3 out of the 80 patients with malignant tumors exhibited elevated serum levels of CEA and CA-50, respectively [121].

Additionally, microRNAs (miRNAs) are small non-coding RNAs implied in the regulation of gene expression in cancer (i.e., enhancing protein-coding oncogenes or inhibiting tumor suppressors). They have emerged as promising circulating biomarkers for diagnostic and prognostic purposes both in HNCs and SGCs; in fact, circulating miRNAs contribute to intercellular signaling and are often dysregulated in different cancer types [122,123,124,125,126,127]. As for SGCs, 95 miRNAs have been assessed in the blood and saliva of patients with SGCs, and it has been shown that in malignant tumors, compared to the control group, plasma miR-30e was significantly upregulated [128]. On the other hand, two studies have reported variations in miRNA expression in saliva samples from patients with malignant or benign parotid gland tumors [129]. Furthermore, a four-miRNA combination (hsa-miR-132, hsa-miR-15b, mmu-miR-140, and hsa-miR-223) was able to distinguish saliva samples of patients with malignant tumors from those with benign parotid gland tumors, achieving a sensitivity of 69% and a specificity of 95% [130]. Another study investigated miRNA expression levels in saliva samples from patients with parotid gland neoplasms versus healthy controls and identified four miRNAs (hsa-miR-296-5p, hsa-miR-1233, hsa-miR-1267, and hsa-miR-1825) that were significantly upregulated in all saliva samples from patients with parotid gland neoplasms. Additionally, three miRNAs (hsa-miR-103a-3p, hsa-miR-211, and hsa-miR-425-5p) were significantly downregulated in tumor patients [131].

In saliva samples, other biomarkers have been found and described. A nuclear magnetic resonance (NMR)-based metabolomic analysis of saliva from patients with SGCs (n = 36) compared to healthy people (n = 23) has been conducted. The findings revealed that individuals with parotid tumors exhibit a distinct metabolomic profile, marked by abnormalities in the concentrations of several amino acids. Notably, alanine and leucine levels were particularly significant, suggesting disruptions in the metabolic pathways of glucogenic amino acids and ketone bodies [132].

A summary of liquid biomarkers is represented in Table 2.

## 4. Precision Oncology in SGCs: A Brief Overview of Emerging Therapeutic Options

The advent of targeted therapies has introduced a new era of precision oncology for SGCs, particularly for aggressive subtypes such as SDC and secretory carcinoma.

Established therapies, such as HER2 inhibitors, have shown efficacy in HER2-positive SGCs, mostly revealed in SDCs (in 30% of cases).

A Japanese phase II trial investigating the combination of trastuzumab and docetaxel in HER2-positive R/M SDCs reported an objective response rate (ORR) of 70% and a clinical benefit rate of 84%, with a median PFS (mPFS) and mOS of 8.9 and 39.7 months, respectively [133].

Beyond trastuzumab, other anti-HER2 agents, such as pertuzumab and Ado-trastuzumab emtansine (T-DM1), have been explored, particularly in cases of disease progression after first-line therapy [134]. More recently, emerging drugs, such as the ADC trastuzumab deruxtecan (T-DXd), have been evaluated. The DESTINY-PanTumor02 trial demonstrated durable clinical benefits for T-DXd in HER2-positive solid tumors, including SGCs, reporting an ORR of up to 61.3% in HER2 IHC 3+ populations (irrespective of prior HER2 therapy), with mOS exceeding 21 months even in heavily pre-treated patients. The greatest benefit was observed in the IHC 3+ subgroup, underscoring the potential role of T-DXd as a tumor-agnostic therapy for HER2-overexpressing solid tumors [135].

A further area of interest includes hormone therapies such as androgen deprivation therapy (ADT), particularly for R/M SDCs, due to the overexpression of AR. However, AR positivity is no longer a sufficient predictive biomarker of efficacy. It has been demonstrated that high levels of SRD5A1 mRNA are strongly predictive of better efficacy in AR-positive SDC patients treated with combined androgen blockade [136]. On the other hand, a high expression of EZH2, a histone methyltransferase of histone H3, and H3K27me3 exhibit a predictive value for poor efficacy of AR-targeted therapy [137].

ADT regimens typically involve non-steroidal anti-androgens, such as bicalutamide, either alone or in combination with luteinizing hormone-releasing hormone (LHRH) analogs.

A prospective phase II study assessed the efficacy and safety of combined androgen blockade with leuprorelin acetate and bicalutamide in SGCs, demonstrating equivalent efficacy and less toxicity compared with conventional chemotherapy in the unresectable, locally advanced or metastatic setting, with an ORR of 42% (11% CR) and an mPFS and an mOS of 8.8 and 30.5 months, respectively [138]. A phase II clinical trial demonstrated ORR of 21% and a disease control rate of 62.5% in AR-positive SGCs treated with abiraterone and an LHRH agonist who progressed on ADT in the second-line setting, with an mPFS of 3.65 months (consistent with the results of second-line chemotherapy) and an mOS of 22.47 months (better than observed with chemotherapy) [139]. However, the final results of EORTC1206 (NCT01969578) showed that ADT (a combination of triptorelin and bicalutamide) did not outperform chemotherapy as a first-line treatment for AR-expressing SDC patients. Interestingly, ADT could also be amenable in poor-risk AR-positive SDC in the adjuvant setting, as demonstrated in a retrospective series [140].

A currently recruiting randomized phase II trial, the DUCT study (NCT05513365), aims to evaluate the benefit of dutasteride in addition to combined androgen blockade in order to overcome therapy resistance in patients with AR-positive R/M SDC previously treated with ADT [141]. Given the rarity of SGCs and the lack of therapeutic options, ADT is a valid option for AR-positive subtypes, warranting further exploration.

The *MYB-NFIB* fusion, a specific genetic hallmark of ACC, has emerged as a promising target for therapy. Preclinical models using all-trans retinoic acid (ATRA) have demonstrated effective suppression of tumor growth and proliferation in ACC patient-derived xenograft models. These findings have led to ongoing clinical trials aimed at validating ATRA’s efficacy in SGC patients [142].

Gamma-secretase inhibitors, which target *NOTCH* mutations frequently found in aggressive ACC, are currently being evaluated in early-phase clinical trials. Preliminary evidence from these studies suggests that such inhibitors may contribute to disease stability, offering a novel approach for this challenging subtype [143,144].

Overexpression of EGFR, observed in 17–100% of SGCs across all histological subtypes (most commonly in ACC, but also MEC and SDC), has been explored as a therapeutic target with controversial results. In SGCs, cetuximab achieved a clinical benefit rate of 50% but failed to determine objective responses in a phase II study [145]. However, case reports have documented CRs or PRs in metastatic SGC patients treated with EGFR inhibitors combined with chemotherapy, highlighting the potential role of such combinations in specific clinical contexts [146,147].

BRAF V600E mutations, known drivers of oncogenesis, detected via NGS or IHC, are occasionally present in SGCs and have shown responsiveness to targeted therapies with BRAF plus MEK inhibitors [148].

The PI3K/AKT/mTOR pathway plays a crucial role in cellular survival, proliferation, and resistance to therapy. Mutations in *PIK3CA* have been identified in various SGC subtypes, particularly in SDC. Preclinical studies and case reports demonstrated tumor responses to PI3K inhibitors, underlining their potential as a therapeutic strategy in this defined molecular subgroup [149,150].

The efficacy of immunotherapy in SGCs remains controversial. High-grade MEC and SDC can be considered to be endowed with an “immune-hot” tumor microenvironment [151]. A retrospective experience showed promising clinical efficacy of PD-1 inhibitors in SGCs (especially in CA ex PA and SDC) [152], whereas pembrolizumab, an anti-PD-1 antibody, alone or in combination with radiotherapy demonstrated unflattering results in a phase II study [153]. These differences can be explained by the complexity of the tumor microenvironment, which is highly variable between different SGC subtypes and deeply influences the response to immunotherapy [154]. However, immune checkpoint inhibitors lack clinical efficacy in SGCs, with an ORR inferior to 20% as monotherapy [153]. The combination of nivolumab, an anti-PD-1, and ipilimumab, an anti-CTLA4, in patients with R/M SGCs showed an ORR of 16%, meeting the primary efficacy endpoint of the study. While the treatment showed limited efficacy in ACC, with few responses, it appeared promising for non-ACC SGCs, especially for SDC [155].

Recently, NTRK inhibitors have been evaluated in the case of TRK fusions. Larotrectinib, a selective TRK inhibitor, has been investigated in patients with SGCs from two clinical trials (NCT02122913 and NCT02576431). Tumor histology consisted of secretory carcinoma in 54%, adenocarcinoma in 21%, and MEC in 13%, with additional cases of ACC, glandular sarcomatoid carcinoma, and adenocarcinoma NOS. All patients exhibited an ETV6-NTRK3 gene fusion [156]. The ORR to larotrectinib was 92%, including 79% partial responses (PRs) and 13% complete responses (CRs). The median response time was 1.84 months (range: 0.99–5.98 months), with treatment durations spanning from 0.95 to over 60.4 months. The PFS rate at 36 months was 66%, while the OS rate was 91%. Moreover, larotrectinib exhibited a favorable safety profile, with predominantly low-grade adverse events and no treatment discontinuations due to drug-related toxicities [156].

Similarly, entrectinib, a pan-TRK inhibitor, has demonstrated efficacy in NTRK fusion-positive tumors. A single case study involving a patient with former MASC reported significant tumor regression following treatment with entrectinib [157].

Both larotrectinib and entrectinib received Food and Drug Administration (FDA) and European Medicine Agency (EMA) tissue-agnostic approval.

Second-generation TRK inhibitors, including selitrectinib (BAY 2731954, LOXO-195) and repotrectinib (TPX-0005), were developed to address on-target resistance mechanisms. Results from a phase I trial of selitrectinib indicated a 45% ORR in patients with confirmed on-target resistance mutations; 10% of cases investigated were secretory carcinoma [158].

While data for repotrectinib remain limited, ongoing phase I/II trials have reported a confirmed partial response in a patient with secretory carcinoma who had progressed on entrectinib [159].

Unremarkable results were obtained from the combination of pembrolizumab and the pan-histone deacetylases (HDAC) inhibitor vorinostat in a recent phase II trial [160]. Although not fully successful in metastatic patients, attempts are being made to move immunotherapy to the neoadjuvant setting: a phase II trial is currently examining the efficacy of neoadjuvant therapy with carboplatin, nab-paclitaxel and the anti-PD-1 antibody toripalimab, in SGCs [NCT04825938].

Recently, a study showed that around 60% of ACCs have high levels of TROP2 expression on IHC, providing the biological rationale for using anti-TROP2 antibody–drug conjugate (ADC) such as Sacituzumab govitecan, widely used in breast cancer and evaluated in HNSCC, as novel potential therapy [161,162].

Overall, these data underline the importance of adequate molecular profiling to suggest new treatment targets and the need to design future clinical trials with the aim of improving the prognosis and survival outcomes of these rare malignancies.

Table 3 summarizes the association between recurrent alterations and targeted therapies in SGCs.

## 5. Conclusions

In conclusion, this review presents an overview of tissue and liquid biomarkers in SGCs. Histopathological features, along with detectable mutations, molecular alterations, and miRNAs, provide valuable insights into the heterogeneous landscape of SGCs, aiding in the diagnosis, prognosis, and development of targeted therapy. The integration of molecular profiling with targeted therapies, such as HER2 inhibitors, NTRK inhibitors, and ADT, underscores the shift towards precision oncology in SGC management.

Liquid biopsy, encompassing ctDNA, CTCs, and exosomes, represents a promising, minimally invasive approach for real-time disease monitoring and personalized therapeutic strategies in clinical oncology, and SGCs are no exception. Although it may not fully replace traditional tissue biopsies at this stage, liquid biopsy offers several advantages, such as less invasive early detection, disease monitoring, and enhanced treatment personalization. The future of liquid biopsy in SGCs will depend on the refinement of analytical techniques, the discovery of more specific biomarkers, and the clinical validation of findings. As research and technology progress, liquid biopsy has the potential to become a key component of the diagnostic and therapeutic approach for patients with these rare and complex tumors.

Despite these advances, the rarity and phenotypic heterogeneity of SGCs necessitate further research to validate biomarkers and optimize treatment strategies. The future lies in well-designed, molecularly stratified clinical trials to improve outcomes for patients with these complex malignancies.

## Figures and Tables

**Table 1:** Classification of recurrent histopathological features and molecular alterations in different salivary gland cancers subtypes *.

Histological Subtype	Histopathological and Immunohistochemical Markers	Detectable Mutations	Molecular Alterations
Mucoepidermoid carcinoma (MEC)	*Positive for p63 or p40 with no detection of S100 or SOX10; however, a subset of solid/trabecular MECs may be completely negative for p63 and p40.*	*CDKN2A* *TP53* *PIK3CA* *HRAS* *POUF6F2*	*CRTC1::MAML2* *CRTC3::MAML2* *EWSR1::POU5F1*
Adenoid cystic carcinoma (ACC)	*Pancytokeratin strong positivity in ductal cells and weaker staining in myoepithelial cells.* *CK7 and c-KIT (CD117) are generally expressed in ductal cells, while p63, p40, calponin, and α-SMA are associated with myoepithelial cells.* *MYB protein overexpression.*	*PIK3CA* *TP53* *NOTCH* *TERT*	*MYB::NFIB* *MYBL1::NFIB* *MYB::PDCD1LG2* *MYB::EFFR3A* *MYBL1::RAD51B* *MYBL1::YTHDF3* *NIB::AIG1*
Acinic cell carcinoma (AciCC)	*Positive for CK7, SOX10, DOG1 and NR4A3 (NOR1) and negative for p40/p63, mammaglobin, and S100.*	*PTEN* *TP53* *CDKN2A/B* *BRAF* *NF1*	*SCPP::NR4A3* *HTN3::MSANTD3* *PON3-LCN1*
Salivary duct carcinoma (SDC)	*CK7 shows consistent positivity, while S100 and SOX10 are negative. Staining for p63 can assist in identifying the intraductal component by highlighting the basal/myoepithelial cells around the neoplastic cells. AR-positive. Strong and diffuse immunoreactivity for HER2 in approximately one-third of SDCs.*	*TP53* *PIK3CA* *PTEN* *HRAS*	*AR gene alterations* *ERBB2 amplifications* *NF1* *KMT2C* *EGFR* *ALK* *CDKN2A* *NOTCH1* *KDM5C* *NRAS* *BRAF* *AKT* *ETV6* *NTRK3*
Secretory carcinoma (SC)	*CK7, S100, SOX10, vimentin, mammaglobin positive;* *p63, p40, NR4A3, and DOG1 negative.* *Pan-TRK positive.*	*-*	*ETV6-NTRK3 fusion* *ETV6-RET* *ETV6-MAML3* *ETV6-MET*
Polymorphous adenocarcinoma (PAC)	*CK-7, S100, CEA, GFAP, SOX10 positive, patchy expression of p63; p40 typically negative.*	*PRKD1* *PTEN* *FGFR1* *TSC2*	*ARID1A::PRKD1* *ARID1A::DDX3X* *PRKD2, PRKD3*
Hyalinizing clear cell carcinoma (HCCC)	*p40 and CK5/6 diffuse positivity; mucin positive; p16 positive with no transcriptionally active HPV. Myoepithelial markers such as S100, SMA, and calponin are negative.*	*-*	*EWSR1::ATF1 fusion* *EWSR1::CREM*
Intraductal carcinoma (IDC)	*Intercalated ducts and oncocytic IDCs exhibit positivity for S100, SOX10, and mammaglobin, but do not express AR or GCDFP-15. In contrast, apocrine IDCs display an inverse staining pattern. Mixed IDCs present a combined immunoprofile. Myoepithelial cells within IDCs consistently stain positive for p40/p63, CK14, SMA, and calponin.*	*PIK3CA* *HRAS* *BRAF p.V600E* *TP53*	*NCOA4::RET* *TRIM33::RET* *TRIM27::RET* *TUT1::ETV5,* *KIAA1217::RET* *STRN::ALK fusion*
Epithelial–myoepithelial carcinoma (EMC)	*Luminal cells typically express CK7, while abluminal cells are generally positive for SMA, calponin, and p63/p40.*	*HRAS* *PLAG1* *HMGA2*	*CTNNB1* *AKT1* *PIK3CA* *FBXW7*
Carcinoma ex-pleomorphic adenoma (CA ex PA)	*PLAG1 and HMGA2 for the identification of the PA component. AR positivity could be indicative of salivary duct CA ex PA.*	*-*	*PLAG1/HMGA2 rearrangements*
Myoepithelial carcinoma (MECA) de novo, MECA ex PA	*Positive for SOX10, S100, and myoepithelial markers such as SMA, calponin, and p63/p40.*	*-*	*PLAG1::FGFR1* *PLAG1::TGFBR3* *EWSR1 rearrangement* *EWSR1-ATF1*

* Non-exhaustive table of histopathological characteristics.

**Table 2:** Liquid biomarkers in salivary gland cancers.

Liquid Biomarker	Description	Origin	Function	References
Circulating tumor DNA (ctDNA)	Fragments of tumor-derived DNA released into bodily fluids; enriched in fragments of 90–150 bp length.	Plasma, saliva	Early diagnostic biomarker, molecular characterization (ctDNA mutation), real-time efficacy monitoring biomarker.	[99,100]
Circulating tumor cells (CTCs)	Malignant cells detached from tumors, traveling in bodily fluids; low abundance in blood (1–10 cells/10 mL).	Peripheral blood	Insights into metastasis, treatment response and surveillance.	[102,103,107,108]
Extracellular vesicles (EVs)	Membrane-bound vesicles (exosomes, microvesicles, apoptotic bodies) containing RNA, proteins, and other biomolecules.	Peripheral blood, saliva	Diagnostic and prognostic potential; modulation of tumor microenvironment and immune response.	[109,110,115,116]
Exosomes	A subtype of EVs (40–150 nm) containing RNAs, miRNA, lncRNA, snoRNA, and proteins.	Peripheral blood, saliva	Diagnostic potential; implicated in tumor progression, modulation of tumor microenvironment; therapeutic targeting.	[118]
Inflammatory markers	Elevated IL-33 and its sST2 receptor levels in parotid gland tumors.	Serum	Differentiation between tumors and healthy controls.	[119]
CEA, CA-50, CA 19-9	Tumor-associated antigens; higher levels in saliva of patients with malignant tumors compared to benign and controls.	Saliva	Salivary antigens levels more sensitive than serum measurements for tumor detection; differentiation between malignant and benign parotid gland tumors.	[120,121]
Circulating miRNAs	Dysregulated miRNAs with specific profiles for benign and malignant tumors; notable miRNAs include miR-21, miR-23a, miR-30e, and others.	Peripheral blood, saliva	Diagnostic and prognostic markers; potential for differentiation between malignant and benign tumors.	[122,128,130,131]
Metabolomic biomarkers	Distinct metabolomic profiles in patients with parotid tumors; altered alanine and leucine levels.	Saliva	Indication of metabolic disruptions; potential diagnostic tool: differentiation between tumors and healthy controls.	[132]

Abbreviations: CEA: carcinoembryonic antigen; CA-50: cancer antigen 50; CA 19-9: cancer antigen 19-9; and miRNAs: microRNAs.

**Table 3:** Main associations of recurrent alterations and targeted therapies for salivary gland cancers.

Biomarker	Targeted Therapy	Cancer Type	References
HER2 overexpression/amplification	Trastuzumab, pertuzumab, T-DM1, T-DXd	SDC	[133,134,135]
AR overexpression	Androgen deprivation therapy (ADT)	SDC	[138,139,140,141]
NOTCH mutations	Gamma-secretase inhibitors	ACC	[143,144]
BRAF V600E mutation	BRAF + MEK inhibitors	SDC	[148]
NTRK fusions	Larotrectinib, entrectinib	Secretory carcinoma	[156,157]

Abbreviations: HER2: human epidermal growth factor receptor 2; AR: androgen receptor; ACC: adenoid cystic carcinoma; and SDC: salivary duct carcinoma.

## Data Availability

No new data were created or analyzed in this study. Data sharing is not applicable to this article.
